# Harnessing the Degloved Palm in Crush Injury Management

**DOI:** 10.7759/cureus.63386

**Published:** 2024-06-28

**Authors:** Devananthan Ilenghoven, Salina Ibrahim, Shah Jumaat Mohd Yussof

**Affiliations:** 1 Plastic and Reconstructive Surgery, Hospital Al Sultan Abdullah, Puncak Alam, MYS; 2 Surgery, Faculty of Medicine, Universiti Teknologi Mara, Sungai Buloh, MYS; 3 Plastic and Reconstructive Surgery, Sungai Buloh Hospital, Sungai Buloh, MYS

**Keywords:** full thickness skin graft, crush avulsion injury, degloved palm, biological dressing, autologous skin

## Abstract

Degloving injuries of the upper limbs, common in industrial settings, pose significant reconstructive challenges. The injury's severity dictates the approach, from primary closure and skin grafting to complex free tissue transfer. Proper preparation of both the wound bed and degloved tissue is crucial, as the degloved tissue can serve as an effective biological dressing. Furthermore, salvaging this tissue and preparing it as a full-thickness skin graft can lead to good graft take-up and healing.

This case report presents a 23-year-old male who sustained a severe crush and degloving injury to his right hand from heavy machinery. Using meticulous debridement and careful preparation of the degloved tissue, we achieved optimal wound management and coverage. This case highlights the critical role of preparation technique in achieving successful outcomes and underscores the potential benefits of using the degloved tissue prior to complex reconstructive scenarios, offering valuable insights for clinical practice.

## Introduction

Degloving injuries are avulsion-type injuries common in extremities or digits. Degloving injury of the palm results in separation at the subcutaneous level, sparing the palmar fascia, retinacular system, and musculoskeletal unit. The palmar skin has multiple perforators running vertically, providing a robust arterial supply to the subepidermal plexus. However, inadequate venous drainage makes it invariably non-viable [1]. Due to severe local tissue injuries, the degloved skin and soft tissue are often effectively dead [2]. Thus, optimal wound bed preparation significantly increases the success of any subsequent soft tissue cover.

The autologous biological dressing is a critical element of wound bed preparation. The use of degloved skin as an autologous biological dressing can provide optimal healing conditions for such injuries. It is a natural product intended for closing the debrided wound during the prolonged healing period while awaiting skin grafting or flap reconstruction. Being superior to synthetic materials and readily available, biological dressing has a more intact extracellular matrix structure that advances wound healing [3]. The degloved skin must be adequately prepared before being applied as a dressing.

Biological dressings generally come in variations including autograft, allograft, and composite graft dressings. Composite grafts are further divided into epidermal and dermal components, dermal replacements, and epidermal grafts [4]. Application of biological dressing is a useful technique for managing difficult wounds where other reconstructive techniques are limited.

## Case presentation

**Figure 1:**
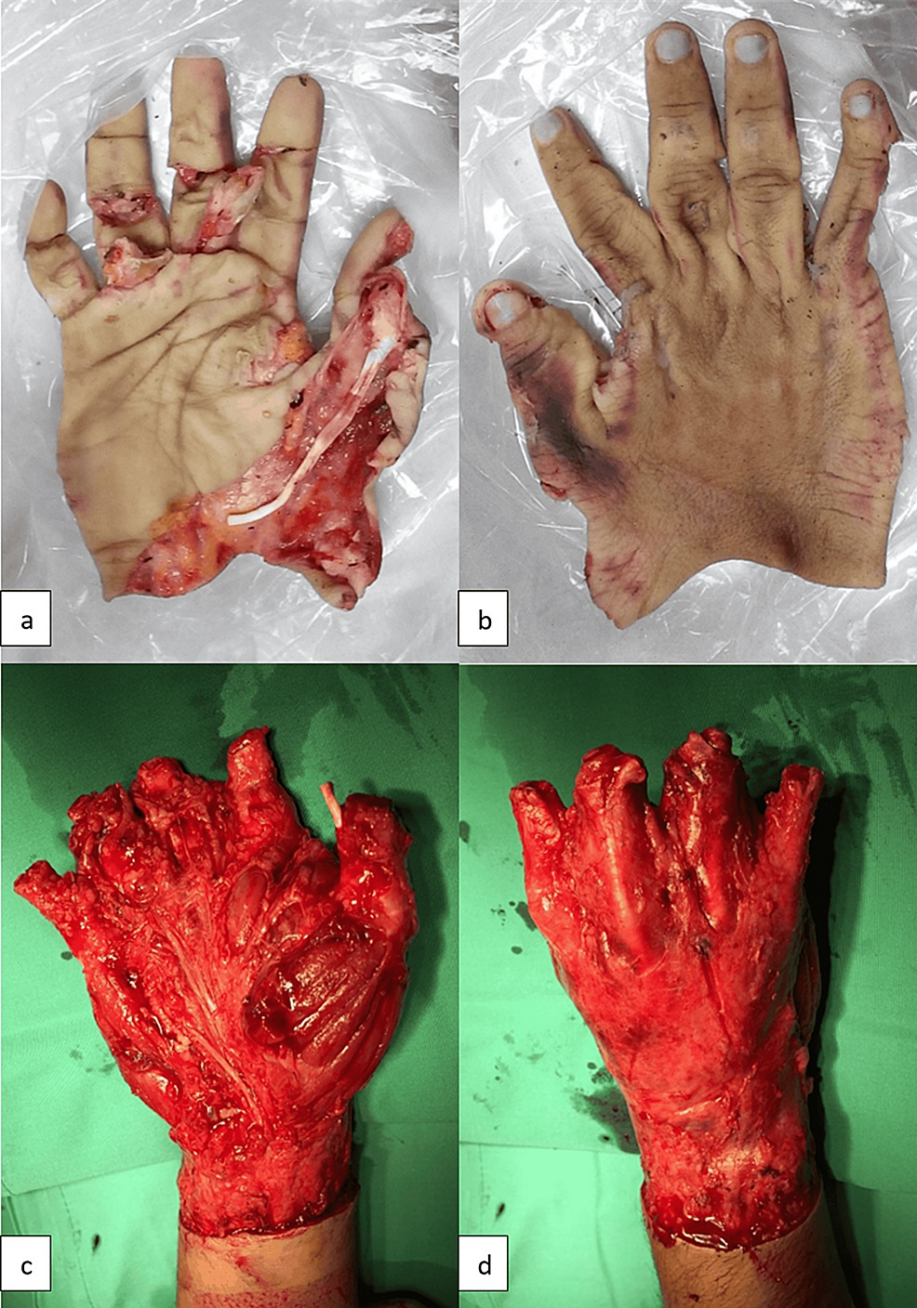
Circumferential degloving skin of right hand down to subcutaneous plane (a,b). Degloved hand up to the proximal right wrist with amputation of all distal phalanges (c,d).

A 23-year-old male arrived at the emergency department of Sungai Buloh Hospital, a tertiary hospital and the state's trauma center, with a crushed and degloved wound on the right hand. His right hand got caught inside heavy machinery, resulting in an avulsion injury to his dominant hand. He is a non-smoker, has no comorbidities, and works as a heavy machinery operator. The degloved skin was placed in a bag of ice, and he was rushed to the hospital. Total ischemic time was three hours. On clinical assessment, the patient had a circumferential degloving injury involving the skin and subcutaneous plane up to the proximal right wrist. Muscles, tendons, and fascia of the right hand were exposed with total amputation of the thumb, index, middle, and ring fingers at the level of the proximal phalanx and total amputation of the little finger at the level of the middle phalanx (Figure 1a-d). He was able to perform active flexion and extension movements at the metacarpophalangeal joint. Replanting was not possible due to the crushing of the avulsed blood vessels with extensive intimal injury and loss of vessel length. He was covered with broad-spectrum antibiotics and given adequate analgesics. Bleeding from the wound was secured with compression dressing in the Emergency Room, and the patient underwent emergency surgery.

**Figure 2:**
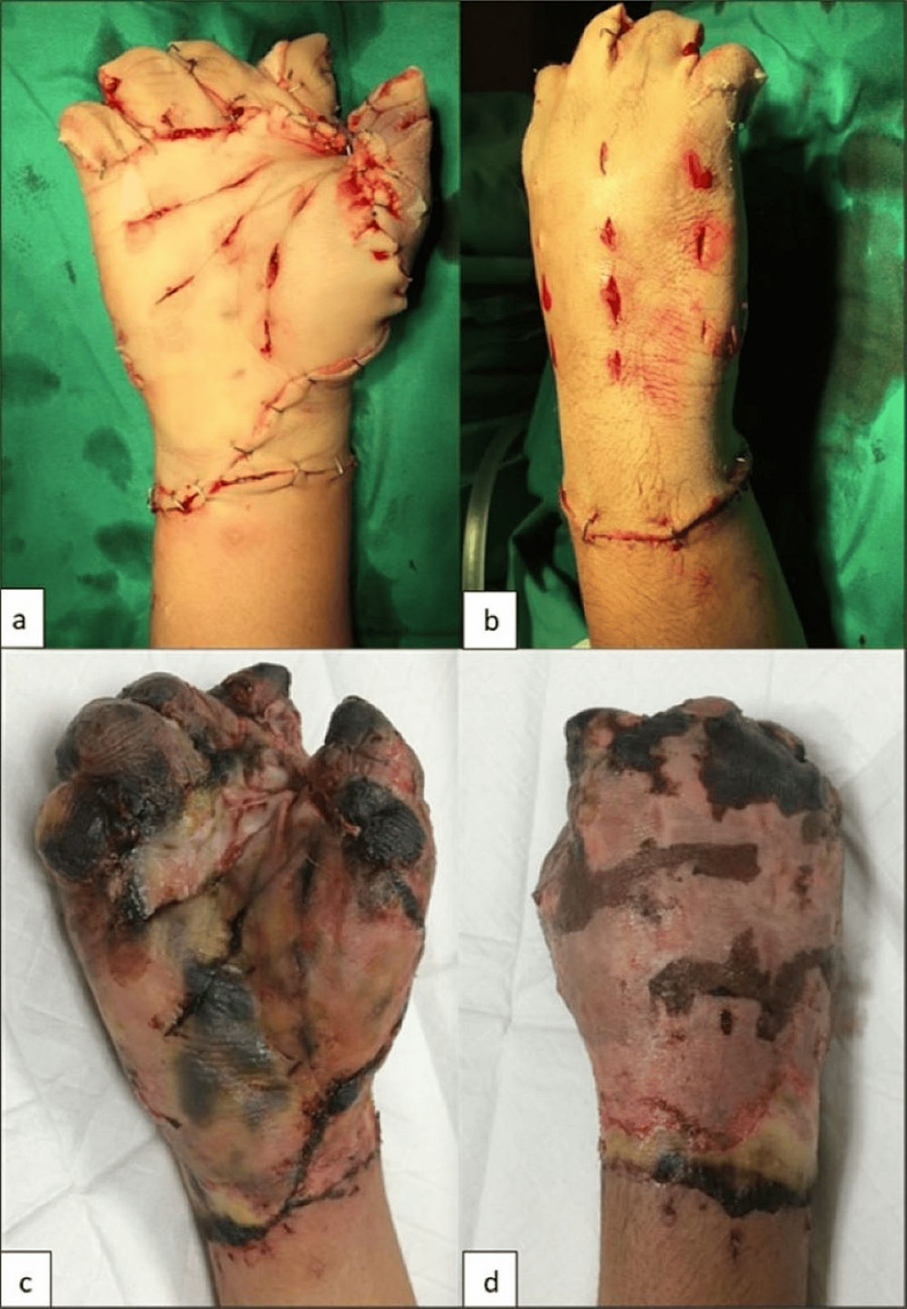
Degloved skin refashioned and applied as full thickness skin graft (a,b). Partial epidermolysis with intact dermis seen on day 4 post operative (c,d).

The degloved tissue was preserved by wrapping it in sterile saline-soaked gauze, placing it in a sealed plastic bag, and refrigerating it at 4°C while waiting for surgery. The tissue was prepared by rinsing with povidone-iodine solution and irrigating with 0.9% saline. The non-viable middle and distal phalanges were amputated, and the remaining degloved skin was refashioned; subcutaneous fat was removed completely, transforming it into a full-thickness skin graft consisting of only dermis and epidermis (Figure 2a,b). The skin was used as a biological dressing. 

**Figure 3:**
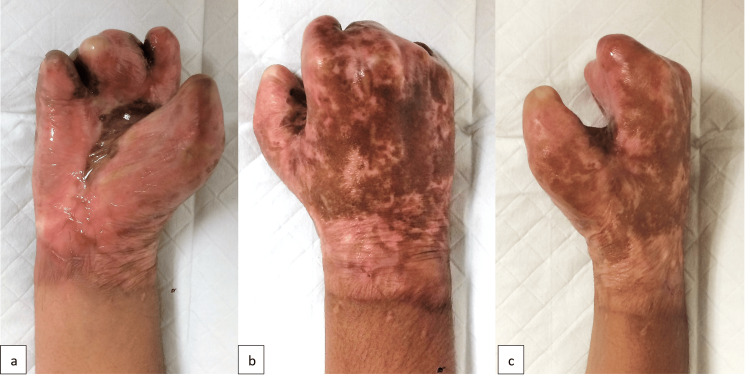
Post web space contracture release and hand function gains (a-c).

Wound inspection on day four showed partial epidermolysis, but the dermis remained intact (Figure 2c,d). Epithelialization occurred, and his wound healed by the third week post-procedure with complete soft tissue coverage. He started strengthening and functional exercises with the physiotherapist in the fourth week and had regular outpatient follow-ups. He was also managed by an occupational therapist with task-oriented training such as holding, grasping, moving, and placing objects. Unfortunately, the patient defaulted on a few follow-ups and gradually developed contracture. He refused the soft tissue reconstruction option to preserve tendon functions. He underwent three more surgeries for contracture release of web spaces with full-thickness skin grafting to regain some hand function (Figure 3a-c). Throughout his follow-up period, he used a resting hand-wrist orthosis with adjustments after every surgery to prevent further contracture.

During his last follow-up, 18 months post initial injury, he was able to use a pen for signatures, but had difficulty gripping a ball. He was able to return to his previous job and was somewhat satisfied with his hand functionality. Further evaluation with the Disabilities of the Arm, Shoulder, and Hand Questionnaire and the Michigan Hand Outcomes Questionnaire (MHQ) scores were 23 and 45, respectively. The MHQ score was broken down by component: (1) Overall hand function: 55; (2) Activities of daily living: 50; (3) Work performance: 35; (4) Pain: 25; (5) Aesthetics: 43; and (6) Satisfaction with hand function: 67.

## Discussion

Trauma leading to degloving injuries is debilitating. Accurate assessment of degloved tissue, preservation of the amputated part, and evaluation of all available surgical options are crucial. Assessment, preservation, and ischemic time play a critical role, and every case should be urgently referred to a center with microsurgical expertise. Surgical options in the hands of an expert include replantation, terminalization of the stump, toe-to-finger transfer, and pollicization of digits [5,6]. The surgery of choice varies from case to case to provide the best outcome for the patient. Emphasis should be placed on high-quality replantation; however, this can be challenging. The patient should be made aware that not all injuries are replantable and offered alternative options. Compared to adults, children have more remarkable functional recovery due to the high potential for nerve regeneration, and replantation should be prioritized. Replantation provides better functional outcomes for holding, catching, and pinching movements [7].

Challenges in performing replantation include (1) availability of expertise in microsurgery, (2) prolonged warm ischemia of more than 12 hours, (3) amputations at multiple levels, (4) long operative times (contraindicated in concurrent life-threatening injuries or patients with multiple significant comorbidities), and (5) high failure rates in patients with obliterative vascular diseases, diabetes mellitus, and smokers [2,8]. Risks of early complications such as infection, thrombosis in the anastomosed vessel, and venous congestion are significant. Subsequent re-exploration surgeries might be necessary to salvage the replanted digits or limb. Late complications such as stiffness, cold intolerance, and tendon adhesions need to be addressed with long-term rehabilitation and secondary surgery.

Degloving injuries that are severely avulsed or crushed are contraindicated for replanting due to poor outcomes. The mechanism of injury, as seen in this case, is an important predictive factor for unsuccessful replantation; hence it is vital to salvage the degloved tissue, particularly the glabrous skin of the palm. The degloved portion of the skin is often neglected or discarded when patients are brought to the hospital. Salvaging this part is beneficial for the patient, especially if it can later provide coverage. Emphasis must be placed on processing the degloved skin and using it for coverage as an autologous biological dressing.

Alternatively, other reconstructive options should be considered for soft tissue coverage to protect the tendon gliding mechanism, which is paramount for hand functioning. A reverse radial forearm pedicle flap is preferred for coverage, offering mobile, thin skin with similar features to the dorsum of the hand. This flap is also more reliable and versatile compared to the posterior interosseous artery flap for degloving hand injuries [9]. Free anterolateral thigh flap, groin flap, or wrap-around abdominal flap are other options; however, they are bulky in appearance and would require subsequent debulking surgery.

Biological dressing is an excellent option for dressing high-grade stress injuries. Dressing options considered here were (1) autologous: patient’s tissue; (2) allograft: donor tissue from the same species, available from human skin banking procedures; and (3) composite dressing: tissue-engineered skin substitutes [10]. We decided to utilize the degloved palm as it was readily available, making it cost-effective. The patient’s own tissue, particularly glabrous skin, is superior to allografts and composite dressing materials as it minimizes scar formation and wound contraction. Early surgery enhances the outcome for the patient, even when replantation is not possible. Shorter hospitalization, reduced pain, and minimized disability can be achieved by utilizing the prepared degloved skin.

Glabrous palm skin presents several advantages over other types of skin and allografts for skin coverage, attributable to its unique histological and biomechanical properties. Characterized by a thick epidermis and a dense, well-organized collagen matrix in the dermis, glabrous skin offers superior resistance to mechanical stress and abrasion, essential for high-friction areas. Its abundant presence of Meissner's corpuscles enhances tactile acuity, facilitating better sensory function. Additionally, the high concentration of eccrine sweat glands in glabrous skin aids in thermoregulation and maintains skin hydration, crucial for graft viability and integration. Compared to allografts, autologous glabrous skin minimizes immunogenic response, reducing the risk of graft rejection and promoting better long-term outcomes. This intrinsic combination of durability, sensory capacity, and reduced immunological complications underscores the efficacy of glabrous palm skin in reconstructive and reparative dermatological applications.

An essential step taken in this case was the preparation of the degloved skin by removing all subcutaneous fat and transforming it into a full-thickness skin graft, which provided an acceptable outcome. The patient's general health status, age, sequence of injury, and management decisions played a significant role in his outcome. A full-thickness graft with complete removal of the poorly vascularized fat layer has a higher probability of survival. Adequate adipose tissue removal reduces the risk of fat necrosis and bacterial contamination. By performing this, we optimize tissue utilization, minimize donor site morbidity, and replace like with like tissue. Factors that played a vital role in this case include: (1) availability of the degloved skin, (2) early transfer to the hospital, (3) early transit to the operating theatre for surgery, (4) performing the procedure in a sterile environment, (5) irrigation of the wound and graft with an antiseptic solution and normal saline, (6) complete removal of fat from the autologous dressing, and (7) early and regular wound inspection.

## Conclusions

The idea of salvaging degloved tissue, especially the glabrous tissue of the palm, should always be an option for replantation. In cases where replantation is not possible, such as in mangled, crushed, or avulsed degloved limbs or digits, early coverage with autologous biological dressing must be considered. The survival of the degloved segment can be enhanced by conversion to a full-thickness graft and used as a biological dressing while awaiting wound bed preparation for soft tissue reconstruction.
